# Variations in Electrocortical Activity due to Surgical Incision in Anaesthetized Cardiac Patients: Electroencephalogram-Based Quantitative Analysis

**DOI:** 10.1155/2020/4643584

**Published:** 2020-02-27

**Authors:** Manpreet Kaur, Neelam Rup Prakash, Parveen Kalra, Goverdhan Dutt Puri, Tanvir Samra, Manoj Goyal

**Affiliations:** ^1^Centre of Excellence (Industrial and Product Design), Punjab Engineering College, Chandigarh, India; ^2^ECE Department, PEC Chandigarh, Chandigarh, India; ^3^Production Engineering Department, PEC Chandigarh, Chandigarh, India; ^4^Anaesthesia Department, PGIMER Chandigarh, Chandigarh, India; ^5^Neurology Department, PGIMER Chandigarh, Chandigarh, India

## Abstract

This study examines the alterations in scalp recorded cortical activity due to surgical incision in anaesthetized cardiac patients using electroencephalogram (EEG) patterns. The primary aim was to compare the changes in electrocortical activity after surgical incision. The secondary aim was to compare the changes in time, frequency, and wavelet domain parameters after loss of consciousness (LoC) and after intubation. Real-time EEG data were recorded from 19 patients undergoing cardiac surgery and signals were quantified with time, frequency, and wavelet domain parameters. An increase in hjorth activity, hjorth complexity, rms value, total band power, relative delta band power, standard deviation and maxima of approximation coefficients (*a*_5_), minima of detail coefficients (*d*_5_, *d*_4_, and *d*_3_) and decrease in hjorth mobility; approximate entropy; relative theta, alpha, and beta band power; specentropy; median, spectral edge, and mean frequency; mean of detail coefficients (*d*_4_); standard deviation of detail coefficients (*d*_5_, *d*_4_, and *d*_3_); maxima of detail coefficients (*d*_5_); and minima of approximation coefficients (*a*_5_) were observed during LoC. Decrease in hjorth activity; hjorth mobility; rms value; total band power; relative theta band power; median frequency; standard deviation of coefficients (*a*_5_, *d*_5_, *d*_4_, and *d*_3_); and maxima of coefficients (*a*_5_, *d*_5_, *d*_4_, and *d*_3_) and increase in hjorth complexity; mean of detail coefficients (*d*_5_); and minima of coefficients (*a*_5_, *d*_5_, *d*_4_, and *d*_3_) were observed after intubation. Significant decrease in hjorth activity; hjorth mobility; total band power; relative alpha band power; specentropy; median and mean frequency; standard deviation and maxima of detail coefficients (*d*_5_, *d*_4_, and *d*_3_) and increase in rms value; relative delta band power; mean of coefficients (*a*_5_ and *d*_5_); and minima of coefficients (*d*_5_, *d*_4_, and *d*_3_) were observed due to surgical incision. It can be concluded that different spectral and temporal parameters of EEG signals are highly sensitive to induction, intubation, and surgical incision which are potentially informative for measuring the depth of anaesthesia or efficacy of anaesthetic agents.

## 1. Introduction

Intraoperative pain assessment is a challenging task. Investigating the electrocortical variations during painful stimuli may help in the development of a monitor or an index for the detection of presence or severity of pain. Alteration in different signal representations of EEG signals has been studied due to induction and noxious pain stimulus (intubation and surgical incision) [1–12]. Decrease in alpha power of temporal, occipital regions due to induction and increase in alpha power of frontal, temporal leads; and decrease in delta power of frontal, central leads due to intubation have been reported [1]. Decrease in approximate entropy [2], spectral edge frequency from 16 Hz to 12 Hz, and beta band power and increase in theta band power and total power have been reported due to induction [3]. Increase of delta and beta2 in early induction period and decrease of alpha1, alpha2, and beta have been reported due to induction [4]. Decrease in relative delta activity; increase in relative theta, alpha, and beta activity during induction; increase in delta activity after induction; decrease in relative theta, alpha, and beta activity during LoC and intubation have been reported [5]. Increase in spindle waves (alpha) at surgical concentrations of anaesthesia; decrease in alpha waves; increase in theta and delta waves at deep levels of induction and burst suppression and then flat EEG at deeper levels have been studied [6]. Disappearance of alpha spindles and decrease in SEF has been reported due to noxious stimuli in case of inadequate analgesia [6]. Variations in BIS and power spectral parameters have been reported due to skin incision [7]. An increase in median and spectral edge frequency and decrease in total power have been reported due to noxious stimulus with scoop dehorning in calves [8]. Noxious stimuli during orthopaedic handlimb surgery in anaesthetized sheep elicit change in haemodynamic responses which were accompanied by either “arousal” or “paradoxical arousal” reaction [9]. Classical arousal pattern has been reported after intubation and incision in anaesthetized adult patients undergoing elective surgery [10]. No variation in high frequencies power, loss of episodic frontal alpha amplitude, and burst suppression patterns have been reported due to surgical incision [11]. Sleigh et al. explored variation in bispectral index, 95% spectral edge frequency, and approximate entropy of electrocortical activity during induction, intubation, surgery, and recovery from induction in patients undergoing minor surgery. Decrease in bispectral index, 95% SEF, and approximate entropy have been observed after induction [13]. Two kinds of responses, i.e., “classical” arousal response (dominance of high frequencies activity) [8, 10] and “paradoxical” arousal response (dominance of low frequency activity) [9, 11], have been reported in anaesthetized adults during intubation and surgical incision. So, the results obtained from previous studies are inconsistent. People from medical community have been using BIS monitors for monitoring the level of sedation. But, now a days, no monitor is available that can monitor the variations in electrocortical activity of brain during different stages of surgery. Moreover, few studies have described the effect of noxious stimuli on electrocortical activity of brain. The objective of this study was thus decided as to investigate the variations in electrocortical responses due to noxious stimuli, i.e., intubation and skin incision in anaesthetized cardiac patients. The contribution of this work over past work is that this work explored variations in different spectral and temporal parameters such as time, frequency, and wavelet domain parameters in patients undergoing major surgery such as coronary artery bypass graft, intracardiac repair, atrial septal defect, ventricular septal defect, and etcetera. This work also investigated the variations in different parameters of EEG signal during surgical incision by comparing the signals during preincision and incision stages.

## 2. Materials and Methods

After getting approval from Institutional Ethics Committee of Post Graduate Institute of Medical Education and Research (PGIMER), Chandigarh, and written informed consent, we performed this observational study in accordance with Helsinki's declaration over a period of 2 months in 34 patients scheduled for cardiac surgery. Inclusion criteria were patients aged between 18 and 80 years and patients having cardiac diseases (valvular, congenital, and coronary artery). Exclusion criteria were patients aged <18 and more than 80 years, pregnancy, and emergency cardiac surgeries.

Continuous EEG recordings were made from patients during baseline, LoC, intubation, and incision events using Clarity's Braintech EEG machine (ACE Medical Equipment, Chandigarh, India), which is based on 10–20 electrode placement system of International federation [14, 15], having a sampling rate of 256 Hz, bandpass-filtered between 0.1 and 70 Hz, and notch-filtered at 50 Hz. Several events like LoC, intubation, and incision were marked manually. Electrodes were placed at *FP*_1_, *FP*_2_, *P*_3_, *P*_4_, *F*_z_, *C*_z_, and *P*_z_ locations. Reference electrodes were placed at two earlobes. Impedance was controlled to be less than 5 kilo ohm. Seven unipolar (*FP*_1_, *FP*_2_, *P*_3_, *P*_4_, *F*_z_, *C*_z_, and *P*_z_) and two bipolar recordings (*F*_z_*C*_z_ and *C*_z_*P*_z_) were computed.

### 2.1. Anaesthesia

In the operation theatre, a 16 G i.v. cannula was inserted and monitors were attached (pulse-oximetry, electrocardiogram, periodic noninvasive blood pressure (S/Anaesthesia monitor, Datex Ohmeda Inc., Madison, WI), and BIS (BIS XP, Aspect Medical Systems, Newton, MA in the S/5 Anaesthesia monitor)). Preinduction arterial line and central line were secured in all patients after giving local anaesthetic and continuous arterial blood pressure and central venous pressure measurements were recorded.

Induction of anaesthesia was performed with propofol using closed loop anaesthesia delivery system (CLADS) to target BIS of 50 and fentanyl 3 mcg/kg was the analgesic used in both groups. Muscular paralysis for tracheal intubation was achieved by vecuronium bromide 0.1 mg/kg. Volume-controlled mechanical ventilation with FiO_2_ 0.5, tidal volume 7-8 ml/kg, positive end-expiratory pressure (PEEP) 5 cm H_2_O, and respiratory rate of 12–14/min with air : oxygen mixture (50 : 50) was initiated to maintain end-tidal carbon dioxide (ETCO_2_) values between 30 and 35 mmHg. Maintenance of anaesthesia was done with propofol infusion using CLADS to target BIS value of 40–60. Fentanyl infusion at 1.0 mcg/kg/h was administered for analgesia.

Fentanyl 1 mcg/kg was administered before sterenotomy. Nasopharyngeal temperature, urinary output, and ETCO_2_ were recorded. CPB was initiated after heparinization and cardioplegia was repeated every 20 minutes. MAP was maintained in the range 50–70 mmHg during CPB. Weaning from CPB was performed in a stepwise manner. Appropriate inotropes adrenaline/noradrenaline/dopamine/milrinone was used to maintain adequate tissue perfusion and cardiac output. CLADS was disconnected after surgery and patients were shifted to postsurgical ICU for mechanical ventilation.

### 2.2. Preprocessing of Raw EEG Data

**Figure 1:**
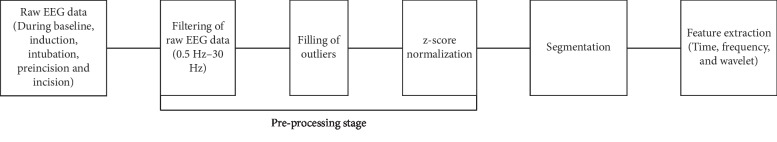
Block diagram of the proposed model.

One minute artefact-free data of baseline, 8 seconds data during LoC, 8 seconds data during intubation, 8 seconds data two minutes prior to incision, and 4 seconds data during incision were used for further analysis. The block diagram of the proposed model is shown in Figure 1.

The raw EEG data were bandpass-filtered between 0.5 Hz and 35 Hz in order to remove the high-frequency noise and low-frequency artefacts. Further, in the filtered data, outliers were replaced by the mean of nearest values. Different algorithms for the normalization of data are available such as “zscore,” “norm,” “scale,” “range,” and “center.” “zscore” method has been used by Sharma et al. for eliminating the subject based bias [16]. Correspondingly, in this work, the data was normalized with the help of z-score normalization method. For the segmentation of EEG signals, four second epochs have been used in previous literature for the segmentation of EEG signals [17, 18]. Normalized data was further segmented into smaller epochs of 4 seconds duration.

### 2.3. Feature Extraction

We computed time, frequency, and wavelet domain parameters from the segmented epochs having time window of 4 seconds during baseline, induction, intubation, preincision, and postincision using matrix laboratory (MATLAB) statistical toolbox as discussed below.

#### 2.3.1. Time Domain

Previous researches have used various time domain features for estimating the effect of induction and nociception. Statistical time domain feature set include approximate entropy, rms value, hjorth parameters (hjorth activity, hjorth mobility, and hjorth complexity), root mean square value, etc.

#### 2.3.2. Frequency Domain

Time domain series having sampling frequency of 256 Hz is processed using fast Fourier transform (FFT) and power spectral density (PSD) and its normalization [19]. The PSD is calculated by computing the fast Fourier transform of autocorrelated sequence and its normalization as shown in equations (1) and (2), respectively:

(1) $$\begin{array}{c} \text{PSD}=\frac{1}{2\pi }\int \left|\text{FFT}{\left(X,t\right)}^{2}\right|\text{d}t, \end{array}$$

(2) $$\begin{array}{c} \text{normalization}=\frac{\left|\text{FFT}{\left(X,t\right)}^{2}\right|}{\max\left(\text{FFT}{\left(X,t\right)}^{2}\right)}. \end{array}$$

Several frequency domain features such as specentropy, relative delta band power (<4 Hz), relative theta band power (4–8 Hz), relative alpha band power (8–15 Hz), and relative beta band power (15–30 Hz) along with mean, median, and 95% spectral edge frequency were computed using FFT. These features have also been used by Panavaranan and Wongsawat for pain state detection [19].

#### 2.3.3. Wavelet Domain

**Figure 2:**
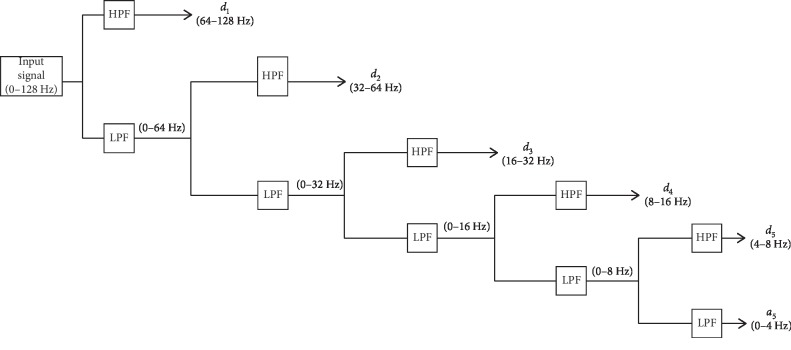
Wavelet decomposition method.

Frequency domain parameters do not contain the temporal distributions of several frequencies. These distributions can be obtained by computing joint time-frequency parameters. In wavelet domain analysis, multiscale feature representation is used and each scale has unique thickness [20]. Each level comprises both down-sampling and filtration stage, i.e., designed using high pass and low pass filters. For computing wavelet domain parameters, the time domain series was firstly decomposed into coarse approximation (*a*_5_) and detail information (*d*_5_-*d*_1_) with Daubechies mother wavelet function. Decomposition was done up to 5 levels (Figure 2). From the decomposed information, mean, standard deviation, minima, and maxima was obtained. As the filtered signal is in the range (0.5–35 Hz), detail coefficients *d*_2_ and *d*_1_ have been ignored.

Different time, frequency, and wavelet domain features were then averaged for baseline, after LoC, postintubation, and postincision for further analysis.

#### 2.3.4. Statistical Analysis

The null hypothesis was taken as “There is no significant variation in electroencephalogram signals recording during baseline and induction; pre- and postintubation; pre- and postincision.” Wilcoxon signed rank test was used to compare the changes in the computed parameters after LoC, intubation, and incision stages. *P* ≤ 0.05 was considered as statistically significant.

## 3. Experimental Results

34 human subjects (patients) undergoing cardiac surgery were recruited. Issues with data recording were encountered in 14 patients, and surgery was deferred in one patient after intraoperative assessment of cardiac functions using TEE by cardiac anaesthetist. Data recording and statistical analysis was done in only 19 patients. The demographic and clinical details of the patient are summarised in Table 1.

### 3.1. Effect of Induction

Due to induction, there are variations in electrocortical activity of the brain. Induction causes significant increase in hjorth activity; hjorth complexity; rms value; total band power; relative delta band power; standard deviation and maxima of approximation coefficients (*a*_5_); and minima of detail coefficients (*d*_5_, *d*_4_, and *d*_3_) and decrease in hjorth mobility; approximate entropy; relative theta, alpha, and beta band power; specentropy; median, spectral edge, and mean frequency; mean of detail coefficients (*d*_4_); standard deviation of detail coefficients (*d*_5_, *d*_4_, and *d*_3_); maxima of detail coefficients (*d*_5_); and minima of approximation coefficients (*a*_5_) at several locations indicated in bold in Table 2.

### 3.2. Effect of Intubation

Due to intubation, significant decrease in hjorth activity; hjorth mobility; rms value; total band power; relative theta band power; median frequency; standard deviation of coefficients (*a*_5_, *d*_5_, *d*_4_, and *d*_3_); and maxima of coefficients (*a*_5_, *d*_5_, *d*_4_, and *d*_3_) and increase in hjorth complexity; mean of detail coefficients (*d*_5_); and minima of coefficients (*a*_5_, *d*_5_, *d*_4_, and *d*_3_) was observed at locations indicated in bold in Table 3.

### 3.3. Effect of Incision

Incision caused significant decrease in hjorth activity; hjorth mobility; total band power; relative alpha band power; specentropy; median and mean frequency; standard deviation and maxima of detail coefficients (*d*_5_, *d*_4_, and *d*_3_) and increase in rms value; relative delta band power; mean of coefficients (*a*_5_ and *d*_5_); and minima of coefficients (*d*_5_, *d*_4_, and *d*_3_) at locations indicated in bold in Table 4.

## 4. Discussion

**Figure 3:**
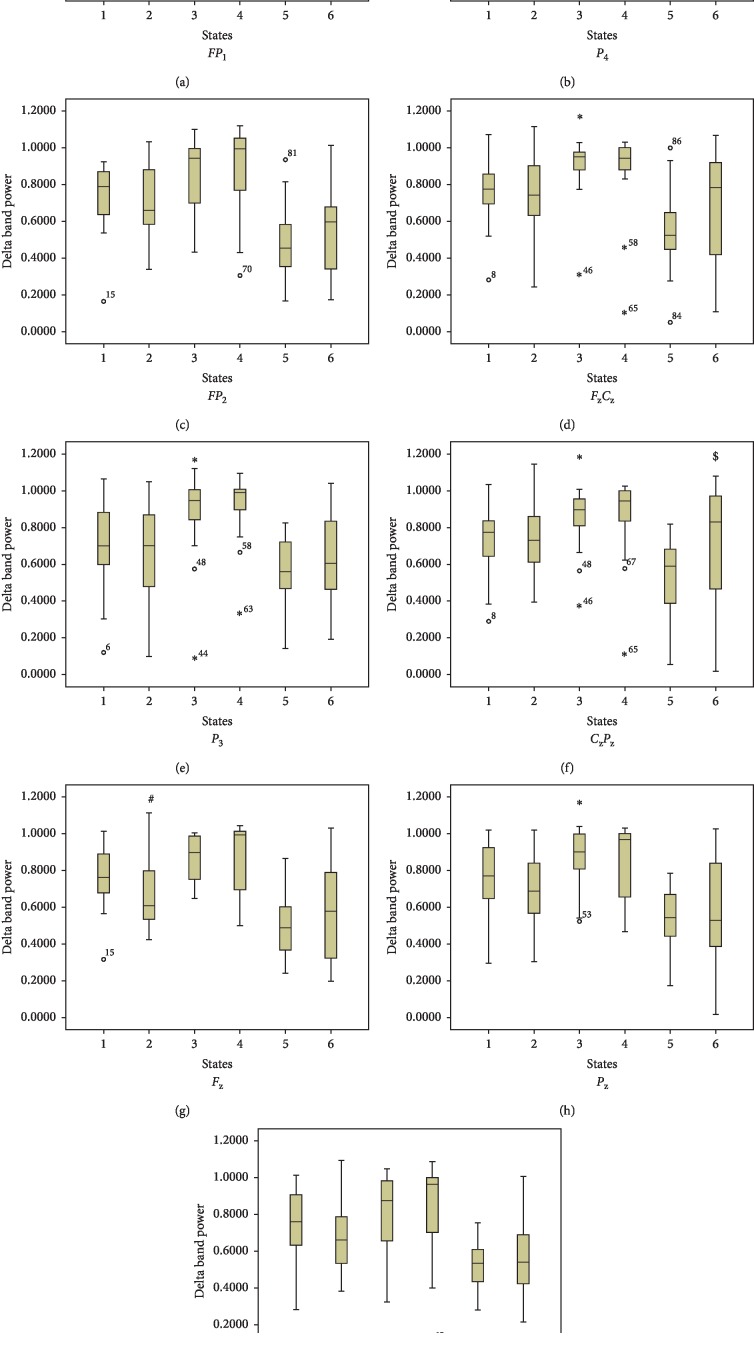
Topography of relative delta band power. Values are medians and interquartile ranges of relative delta power. *X*-axis: 1-normal resting state; 2-start of induction; 3-LoC; 4-postintubation; 5-preincision; 6-postincision. *Y*-axis: relative delta band power. ^*∗*^*P* ≤ 0.05 for baseline vs. LoC; ^#^*P* ≤ 0.05 for baseline vs. during induction; ^$^*P* ≤ 0.05 for preincision vs. postincision.

**Figure 4:**
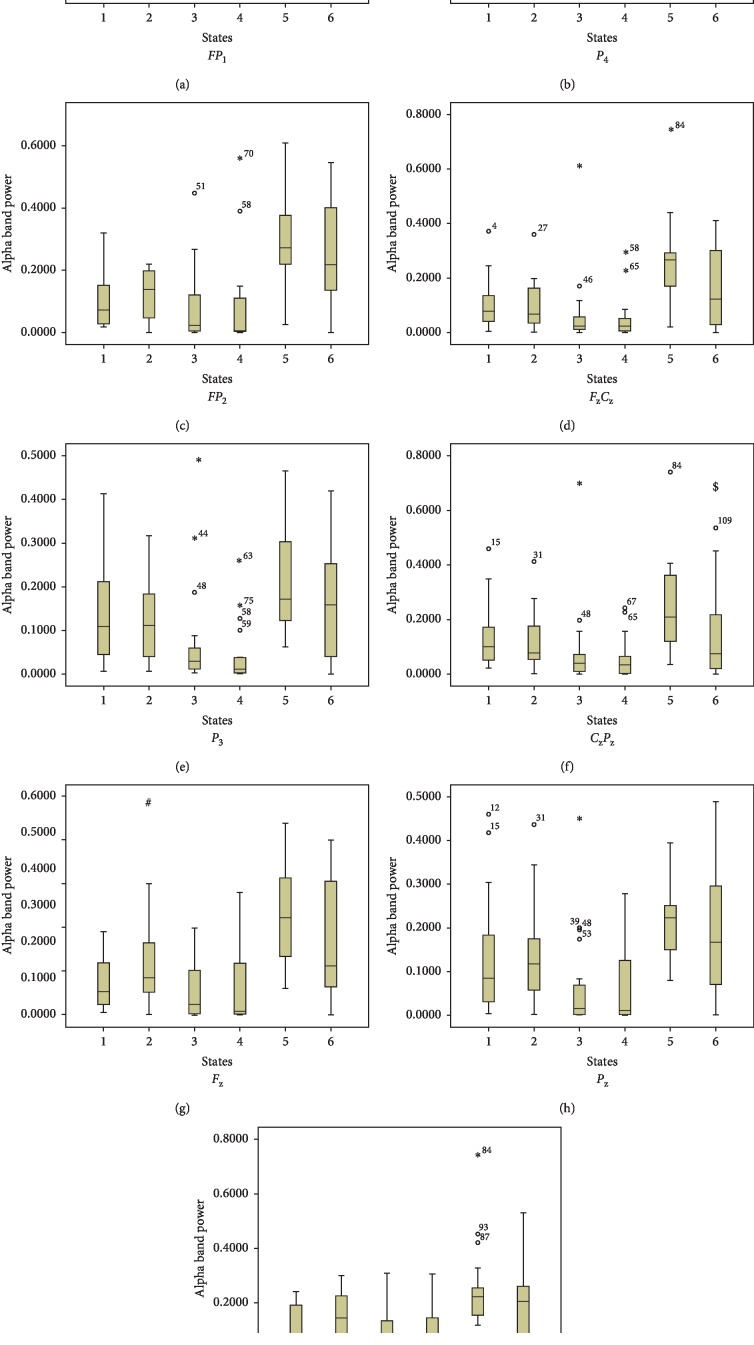
Topography of relative alpha band power. Values are medians and interquartile ranges of relative delta power. *X*-axis: 1-normal resting state; 2-start of induction; 3-LoC; 4-postintubation; 5-preincision; 6-postincision. *Y*-axis: relative alpha band power. ^*∗*^*P* ≤ 0.05 for baseline vs. LoC; ^#^*P* ≤ 0.05 for baseline vs. during induction; ^$^*P* ≤ 0.05 for preincision vs. postincision.

This study showed that due to surgical incision, significant decrease in hjorth activity; hjorth mobility; total band power; relative alpha band power; specentropy; median and mean frequency; standard deviation; and maxima of detail coefficients (*d*_5_, *d*_4_, and *d*_3_) and increase in rms value; relative delta band power; and mean of coefficients (*a*_5_ and *d*_5_); minima of coefficients (*d*_5_, *d*_4_, and *d*_3_) was observed. Induction causes an increase in hjorth activity; hjorth complexity; rms value; total band power; relative delta band power; standard deviation and maxima of approximation coefficients (*a*_5_); and minima of detail coefficients (*d*_5_, *d*_4_, and *d*_3_) and decrease in hjorth mobility; approximate entropy; relative theta, alpha, and beta band power; specentropy; median, spectral edge, and mean frequency; mean of detail coefficients (*d*_4_); standard deviation of detail coefficients (*d*_5_, *d*_4_, and *d*_3_); maxima of detail coefficients (*d*_5_); minima of approximation coefficients (*a*_5_). Induction of anaesthesia causes an increase in low frequency activity which is an indicator of deepening of anaesthesia [1, 4]. Decrease in mean, median, and 95% spectral edge frequency due to signal transition from high-frequency components to low-frequency components was observed, as has been reported during the assessment of depth of anaesthesia with isoflurane or propofol in previous studies [2, 3]. Schwender et al. reported decrease in spectral edge frequency from 16 to 12 Hz during induction and an increase in spectral edge frequency from 12 to 18 Hz during emergence from general anaesthesia. Increase in hjorth complexity signifies the signal similarity to a pure sinusoidal wave and decrease in hjorth mobility, approximate entropy, and specentropy signifies reduction in signal complexity and more similarity, which are indirect indicator of LoC. Decrease in approximate entropy has been reported in EEG signals by Bruhn et al. during the measurement of anaesthetic drug effect with desflurane anaesthesia [2]. We observed a biphasic EEG response in all locations except *FP*_1_ at the time when the patient was being induced as shown in Figures 3 and 4. This decrease in low-frequency delta activity and increase in high-frequency activity (biphasic response) have been reported in past studies [5]. Surprisingly, Rundshagen et al. did not report this kind of variation in EEG signal during induction. This could be possible that this effect is only seen due to slow variation in plasma drug concentration [1].

After intubation, hjorth activity; hjorth mobility; rms value; total band power; relative theta band power; median frequency; standard deviation of coefficients (*a*_5_, *d*_5_, *d*_4_, and *d*_3_); and maxima of coefficients (*a*_5_, *d*_5_, *d*_4_, and *d*_3_) decreased and hjorth complexity; mean of detail coefficients (*d*_5_); minima of coefficients (*a*_5_, *d*_5_, *d*_4_, and *d*_3_) increased. This variation in different parameters signifies further decrease in complexity and high-frequency component in signal. In this study, we did not report cortical arousal due to intubation which is in accordance with the past study [12].

Hung et al. used EEG signals recorded from bipolar location *C*_3_*P*_3_ for assessing anaesthesia depth and relating thiopental concentration with movement. Surprisingly, Rundshagen et al. demonstrated that by using thiopental and fentanyl, classical arousal is not blocked during induction. They used EEG signals recorded from 8 bipolar locations, to map the variations in electrical activity of brain during induction using thiopental/fentanyl and intubation of trachea [1].

After skin incision, the most dominant changes were increase in relative delta power and decrease in relative alpha power. These are an indication of paradoxical arousal, as reported in previous studies [6, 9]. Sleigh et al. reported transition from burst suppression patterns to delta wave component due to surgical incision in propofol or desflurane anaesthetized patients. Decrease in specentropy at *P*_3_ location and decrease in hjorth mobility, median, and mean frequency at *C*_z_*P*_z_ location signifies more similarity and transition from high-frequency component to low-frequency component. These are an indirect indicator of paradoxical arousal. These features may be used further for developing of an index or a monitor for the detection of presence or severity of pain.

Both kind of responses to noxious stimuli, “paradoxical” signifying dominance of low-frequency delta activity as well as “classical” signifying the dominance of high-frequency activity have been reported in the literature. The variation among our results and those past literatures might be elucidated by alterations in (a) level of anaesthesia at the time of intubation and incision; (b) montage used for recording the EEG signals; or (c) effect of anaesthetics on electrocortical activity of brain.

## 5. Conclusion

We concluded that different frequency activities of EEG signal were sensitive parameters of electrocortical brain activity during induction of anaesthesia and noxious stimulation, i.e., intubation and skin incision. EEG waves became more similar during LoC state with increase in low-frequency delta activity and decrease in high-frequency activity. The effect of intubation on EEG was quite small as compared to induction. Due to incision, paradoxical arousal response was observed in EEG patterns.

## Figures and Tables

**Table 1:** Demographic table and clinical details of patients.

Demographic and clinical parameters	Value
Age (years)	47.63 ± 19.13
Sex (M : F)	12 : 7
Weight (Kg)	56.89 ± 15.47
Height (cm)	155 ± 25.9
BMI (Kg/m^2^)	30.06 ± 35.54
Diagnosis	
CCHD	1
Valvular heart disease	7
CAD	7
ACHD	3
Aorta carctation	1
Others	1
Surgical procedures	
ICR	1
Valve repair	8
CABG	7
ASD	2
VSD	1
Thrombectomy	1

**Table 2:** *P* values for statistical significance of comparison of EEG data under baseline vs. LoC.

Features	*FP*_1_	*FP*_2_	*P*_3_	*P*_4_	*F*_z_ * C*_z_	*C*_z_ * P*_z_	*F*_z_	*C*_z_	*P*_z_
Baseline-induction									
Hjorth activity	0.099	0.084	0.314	0.277	**0.04**	0.159	**0.001**	0.421	**0.024**
Hjorth mobility	**0.02**	0.077	**0.016**	**0.001**	**0.018**	0.064	**0.027**	0.295	0.053
Hjorth complexity	**0.024**	0.077	0.277	**0.044**	**0.022**	0.091	0.227	0.212	0.445
Approximate entropy	**0.024**	**0.036**	**0.006**	**0.003**	**0.007**	**0.011**	**0.024**	0.077	**0.018**
RMS value	0.099	**0.009**	0.355	0.053	**0.02**	0.084	**0.001**	0.469	**0.014**
Total band power	**0.04**	0.243	0.376	0.398	0.171	0.398	**0.004**	0.601	**0.016**
Delta band power	**0.024**	0.053	**0.009**	**0.001**	**0.004**	**0.003**	0.064	0.277	**0.044**
Theta band power	**0.008**	**0.004**	**0.016**	**0.003**	**0.002**	**0.016**	**0.006**	0.376	0.314
Alpha band power	0.077	0.398	**0.005**	**0.001**	**0.005**	**0.002**	0.295	0.376	**0.02**
Beta band power	**0.02**	0.053	**0.036**	**0.001**	**0.008**	**0.049**	**0.044**	0.494	0.107
Specentropy	**0.022**	**0.02**	**0.044**	0.107	**0.022**	0.099	0.099	0.26	0.184
Median frequency	**0.049**	0.126	**0.036**	**0.001**	**0.004**	**0.007**	**0.02**	0.573	0.147
Spectral edge frequency	**0.033**	0.064	**0.016**	**0.002**	**0.011**	**0.03**	**0.033**	0.136	0.053
Mean frequency	**0.02**	0.084	**0.027**	**0.001**	**0.004**	**0.006**	**0.036**	0.355	**0.049**
Mean of approximation coefficients (*a*_5_)	0.277	0.469	0.573	0.904	0.841	0.936	0.601	0.334	0.26
Mean of detail coefficients (*d*_5_)	0.658	0.26	0.126	0.084	0.314	0.573	0.573	0.546	0.52
Mean of detail coefficients (*d*_4_)	0.053	**0.027**	0.904	0.809	0.077	0.099	0.136	1	0.936
Mean of detail coefficients (*d*_3_)	0.445	1	0.687	0.421	0.936	0.936	0.26	0.421	0.52
Standard deviation of approximation coefficients (*a*_5_)	**0.02**	**0.005**	0.059	**0.004**	**0.003**	0.059	**0**	0.355	**0.014**
Standard deviation of detail coefficients (*d*_5_)	**0.009**	**0.014**	0.243	0.136	0.198	0.904	0.445	0.717	0.872
Standard deviation of detail coefficients (*d*_4_)	0.053	0.171	0.099	**0.027**	0.117	0.212	0.494	0.778	0.334
Standard deviation of detail coefficients (*d*_3_)	**0.049**	0.126	**0.033**	**0.003**	0.059	0.243	0.212	0.376	0.376
Maxima of approximation coefficients (*a*_5_)	0.184	0.059	0.053	**0.011**	**0.005**	**0.024**	**0.001**	0.398	**0.01**
Maxima of detail coefficients (*d*_5_)	**0.005**	**0.005**	0.159	0.126	0.687	0.968	0.277	0.546	0.778
Maxima of detail coefficients (*d*_4_)	0.077	0.198	0.334	0.099	0.546	0.748	0.872	0.629	0.936
Maxima of detail coefficients (*d*_3_)	0.064	0.117	0.107	0.077	0.07	0.968	0.355	0.841	0.872
Minima of approximation coefficients (*a*_5_)	0.053	0.687	**0.036**	**0.018**	**0.011**	0.059	**0.003**	0.573	**0.022**
Minima of detail coefficients (*d*_5_)	**0.024**	0.053	0.469	**0.033**	0.171	0.184	0.398	0.494	0.841
Minima of detail coefficients (*d*_4_)	**0.04**	0.212	0.117	0.117	0.243	0.809	0.494	0.52	0.748
Minima of detail coefficients (*d*_3_)	0.07	0.107	0.227	**0.018**	0.136	0.904	0.277	0.687	0.573

(*P* ≤ 0.05 was considered as statistically significant).

**Table 3:** *P* values for statistical significance of comparison of EEG data under Preintubation vs. postintubation.

Features	*FP*_1_	*FP*_2_	*P*_3_	*P*_4_	*F*_z_ * C*_z_	*C*_z_ * P*_z_	*F*_z_	*C*_z_	*P*_z_
Pre-post intubation									
Hjorth activity	0.099	0.077	0.314	0.277	**0.04**	0.147	**0.001**	0.421	**0.022**
Hjorth mobility	0.936	0.421	**0.01**	**0.036**	0.778	**0.027**	0.494	**0.044**	0.159
Hjorth complexity	0.355	0.184	0.147	0.147	0.601	0.314	**0.014**	0.421	**0.036**
Approximate entropy	0.717	0.809	0.084	0.084	0.376	0.243	0.658	0.117	0.227
RMS value	0.117	0.159	0.421	0.295	0.064	0.198	**0.001**	0.52	**0.033**
Total band power	**0.049**	0.07	0.171	0.171	**0.04**	0.147	**0.001**	0.243	**0.005**
Delta band power	0.573	0.091	0.212	0.212	0.601	0.227	0.469	0.171	0.778
Theta band power	0.748	**0.008**	**0.03**	0.314	0.421	0.421	0.314	0.295	0.334
Alpha band power	0.184	0.629	0.212	0.184	0.841	0.841	0.872	0.601	0.778
Beta band power	0.064	0.968	0.147	0.334	0.295	0.243	0.872	0.809	0.376
Specentropy	0.687	0.295	0.227	0.295	0.52	0.07	0.936	0.059	0.904
Median frequency	0.184	**0.044**	**0.003**	0.184	0.314	**0.04**	0.376	0.077	0.126
Spectral edge frequency	0.52	0.117	0.117	0.091	0.904	0.243	0.171	0.748	0.398
Mean frequency	0.968	0.084	0.07	0.117	0.968	0.099	0.376	0.171	0.227
Mean of approximation coefficients (*a*_5_)	0.277	0.469	0.573	0.904	0.841	0.936	0.601	0.334	0.26
Mean of detail coefficients (*d*_5_)	0.184	1	**0.008**	0.573	0.334	0.295	0.52	0.295	0.494
Mean of detail coefficients (*d*_4_)	0.26	0.198	0.748	0.778	0.573	0.445	0.717	0.601	0.936
Mean of detail coefficients (*d*_3_)	0.717	0.841	0.212	0.872	0.936	0.778	0.936	0.968	0.872
Standard deviation of approximation coefficients (*a*_5_)	0.184	0.334	0.968	0.629	**0.04**	0.277	**0.005**	0.717	0.159
Standard deviation of detail coefficients (*d*_5_)	**0.016**	**0.033**	**0.03**	0.053	0.171	0.053	**0**	0.198	**0.001**
Standard deviation of detail coefficients (*d*_4_)	**0.04**	0.059	**0.005**	**0.049**	0.227	0.077	**0.002**	0.084	**0**
Standard deviation of detail coefficients (*d*_3_)	**0.044**	0.077	**0.002**	**0.008**	0.314	0.091	**0.005**	0.243	**0.003**
Maxima of approximation coefficients (*a*_5_)	0.184	0.099	0.778	0.658	**0.01**	0.277	**0**	0.546	**0.022**
Maxima of detail coefficients (*d*_5_)	**0.007**	**0.009**	0.099	0.053	0.077	0.077	**0**	0.184	**0.003**
Maxima of detail coefficients (*d*_4_)	**0.036**	0.198	**0.007**	**0.02**	0.053	**0.033**	**0.001**	0.053	**0.007**
Maxima of detail coefficients (*d*_3_)	0.091	**0.04**	**0.011**	**0.02**	0.212	0.077	**0.006**	0.212	**0.005**
Minima of approximation coefficients (*a*_5_)	0.091	0.936	0.629	0.494	0.064	0.494	**0.003**	0.748	0.136
Minima of detail coefficients (*d*_5_)	**0.016**	0.091	**0.014**	0.077	0.198	**0.033**	**0.001**	**0.04**	**0.004**
Minima of detail coefficients (*d*_4_)	**0.03**	**0.005**	**0.004**	**0.036**	0.147	**0.024**	**0.002**	**0.022**	**0**
Minima of detail coefficients (*d*_3_)	0.159	0.099	**0.01**	**0.024**	0.421	**0.008**	**0.02**	0.07	**0.006**

(*P* ≤ 0.05 was considered as statistically significant).

**Table 4:** *P* values for statistical significance of comparison of EEG data under preincision vs. postincision.

Features	*FP*_1_	*FP*_2_	*P*_3_	*P*_4_	*F*_z_ * C*_z_	*C*_z_ * P*_z_	*F*_z_	*C*_z_	*P*_z_
Pre-post incision									
Hjorth activity	0.968	**0.02**	0.07	0.809	**0.009**	0.126	0.421	0.227	0.334
Hjorth mobility	0.469	0.904	0.171	0.227	0.147	**0.02**	0.376	0.084	0.601
Hjorth complexity	0.243	0.355	0.717	0.573	0.421	0.059	0.687	0.334	0.52
Approximate entropy	0.841	0.904	0.376	0.936	0.546	0.147	0.277	0.355	0.469
RMS value	**0.04**	0.212	0.872	0.243	0.07	0.52	0.809	0.398	0.687
Total band power	0.748	**0.018**	0.355	0.778	**0.044**	0.212	0.968	0.398	0.841
Delta band power	0.778	0.376	0.295	0.421	0.091	**0.014**	0.314	0.421	0.658
Theta band power	0.841	0.198	0.295	0.841	0.126	0.053	0.117	0.295	0.687
Alpha band power	0.717	0.601	0.398	0.126	0.07	**0.011**	0.52	0.314	0.26
Beta band power	0.398	0.546	0.445	0.717	0.629	0.212	0.809	0.809	0.687
Specentropy	1	0.872	**0.044**	0.52	0.398	0.376	0.809	0.398	0.376
Median frequency	0.904	0.601	0.147	0.334	0.136	**0.01**	0.314	0.445	0.601
Spectral edge frequency	0.445	0.546	0.398	0.968	0.398	0.117	0.421	0.295	0.872
Mean frequency	0.421	0.601	0.243	0.494	0.159	**0.011**	0.421	0.398	0.601
Mean of approximation coefficients (*a*_5_)	0.872	0.778	0.841	**0.027**	0.658	0.717	0.314	0.778	0.872
Mean of detail coefficients (*d*_5_)	0.198	0.546	0.227	0.295	0.573	0.936	**0.018**	0.064	0.171
Mean of detail coefficients (*d*_4_)	0.355	0.936	0.227	1	0.748	0.872	0.334	0.107	0.159
Mean of detail coefficients (*d*_3_)	0.841	0.52	0.936	0.295	0.717	0.809	0.52	0.778	0.546
Standard deviation of approximation coefficients (*a*_5_)	0.147	0.809	0.809	0.184	0.904	0.494	0.421	0.601	0.687
Standard deviation of detail coefficients (*d*_5_)	0.295	0.212	0.295	0.52	0.227	**0.008**	0.355	0.546	0.243
Standard deviation of detail coefficients (*d*_4_)	0.809	0.573	0.126	0.26	0.107	**0.006**	0.469	0.171	0.573
Standard deviation of detail coefficients (*d*_3_)	0.658	0.601	0.091	0.334	0.198	**0.013**	0.717	0.227	0.494
Maxima of approximation coefficients (*a*_5_)	0.077	0.277	0.52	0.159	0.748	0.904	0.52	0.421	0.809
Maxima of detail coefficients (*d*_5_)	0.778	0.147	0.243	0.573	0.184	**0.007**	0.295	1	0.159
Maxima of detail coefficients (*d*_4_)	0.717	0.494	**0.027**	0.212	**0.033**	0.126	0.355	0.107	0.227
Maxima of detail coefficients (*d*_3_)	0.601	0.159	0.227	0.445	0.314	**0.004**	0.658	0.872	0.872
Minima of approximation coefficients (*a*_5_)	0.936	0.841	0.968	0.421	0.748	0.601	0.445	0.809	0.376
Minima of detail coefficients (*d*_5_)	1	0.126	0.147	0.295	0.421	**0.036**	0.421	0.687	0.277
Minima of detail coefficients (*d*_4_)	0.968	0.376	0.212	0.629	**0.044**	**0.001**	0.295	**0.044**	0.147
Minima of detail coefficients (*d*_3_)	0.748	0.398	0.494	0.376	0.295	**0.04**	0.243	0.629	0.469

(*P* ≤ 0.05 was considered as statistically significant).

## Data Availability

The data used to support the findings of this study are currently under embargo while the research findings are commercialized. Requests for data, after publication of this article, will be considered by the corresponding author.
